# METHYLATION RISK SCORES ARE ASSOCIATED WITH CARDIOVASCULAR RISK FACTORS IN OLDER US ADULTS

**DOI:** 10.1093/geroni/igad104.2772

**Published:** 2023-12-21

**Authors:** Lisha Lin, Wei Zhao, Kira Birditt, Jennifer Smith

**Affiliations:** University of Michigan School of Public Health, Ann Arbor, Michigan, United States; Institute for Social Research, University of Michigan, Ann Arbor, Michigan, United States; Institute for Social Research, University of Michigan, Ann Arbor, Michigan, United States; University of Michigan School of Public Health, Ann Arbor, Michigan, United States

## Abstract

DNA methylation risk scores (MRS) are biomarkers that may prove clinically useful by enabling earlier prevention and individualized treatment of cardiovascular diseases (CVD). However, it is unclear whether relationships between methylation and CVD risk factors differ by demographics or health behaviors. Using existing epigenome-wide association studies, we created MRSs for eight CVD risk factors: systolic and diastolic blood pressure, body mass index (BMI), C-reactive protein (CRP), low- and high-density lipoprotein cholesterols (LDL-C, HDL-C), triglycerides (TG), and fasting glucose. Methylation was measured in whole blood samples from Health and Retirement Study participants (N=3,996, mean age=69.5 years). In linear regression, each MRS was positively associated with its corresponding CVD risk factor after controlling for demographics and health behaviors (all p< 0.001). Adding interaction terms between each MRS and age, gender, or alcohol use revealed differences in MRS associations with their corresponding CVD risk factors across groups: higher MRSs were associated with greater increases in BMI, HDL-C, and TG in younger participants than older participants, greater increases in BMI and HDL-C in females than males, and a greater increase in CRP in non-drinkers than heavy drinkers (≤65 years old: >2 per day for males and >1 per day for females; >65 years old: >1 per day for both genders). In conclusion, some MRSs for CVD risk factors have stronger associations in younger adults, women, and non-drinkers. Additional research is needed to better understand the potential clinical utility of MRSs for early CVD prevention and intervention.

